# Feed-forward and feedback projections of midbrain reticular formation neurons in the cat

**DOI:** 10.3389/fnana.2013.00055

**Published:** 2014-01-10

**Authors:** Eddie Perkins, Paul J. May, Susan Warren

**Affiliations:** ^1^Department of Neurobiology and Anatomical Sciences, University of Mississippi Medical CenterJackson, MS, USA; ^2^Department of Neurosurgery, University of Mississippi Medical CenterJackson, MS, USA; ^3^Department of Ophthalmology, University of Mississippi Medical CenterJackson, MS, USA; ^4^Department of Neurology, University of Mississippi Medical CenterJackson, MS, USA

**Keywords:** oculomotor, head movement, gaze, superior colliculus, reticular formation

## Abstract

Gaze changes involving the eyes and head are orchestrated by brainstem gaze centers found within the superior colliculus (SC), paramedian pontine reticular formation (PPRF), and medullary reticular formation (MdRF). The mesencephalic reticular formation (MRF) also plays a role in gaze. It receives a major input from the ipsilateral SC and contains cells that fire in relation to gaze changes. Moreover, it provides a feedback projection to the SC and feed-forward projections to the PPRF and MdRF. We sought to determine whether these MRF feedback and feed-forward projections originate from the same or different neuronal populations by utilizing paired fluorescent retrograde tracers in cats. Specifically, we tested: 1. whether MRF neurons that control eye movements form a single population by injecting the SC and PPRF with different tracers, and 2. whether MRF neurons that control head movements form a single population by injecting the SC and MdRF with different tracers. In neither case were double labeled neurons observed, indicating that feedback and feed-forward projections originate from separate MRF populations. In both cases, the labeled reticulotectal and reticuloreticular neurons were distributed bilaterally in the MRF. However, neurons projecting to the MdRF were generally constrained to the medial half of the MRF, while those projecting to the PPRF, like MRF reticulotectal neurons, were spread throughout the mediolateral axis. Thus, the medial MRF may be specialized for control of head movements, with control of eye movements being more widespread in this structure.

## INTRODUCTION

The midbrain reticular formation (MRF) has been implicated as an intermediary in the brainstem circuits underlying the control of the eye and head components associated with a gaze change. Physiological support for this contention came initially from experiments in which electrical stimulation of the MRF in macaque monkeys produced horizontal contraversive saccades (Bender and Shanzer, 1964; Cohen and Büttner-Ennever, 1984; Cohen et al., 1985). These stimulation studies defined a caudal MRF region, the central mesencephalic reticular formation (cMRF), which was primarily concerned with the horizontal component of saccades. This was differentiated from more rostral midbrain regions like the interstitial nucleus of Cajal (InC) and rostral interstitial nucleus of the medial longitudinal fasciculus (riMLF), whose main roles include controlling vertical components of conjugate eye movements (Büttner et al., 1977; King and Fuchs, 1979; Crawford et al., 1991; Fukushima et al., 1995). More recently, a region of the MRF adjacent to the InC, the peri-InC portion of the MRF (piMRF), has been associated with vertical gaze components in monkeys (Waitzman et al., 2000b). Several investigations have recorded from neurons within the macaque cMRF and have confirmed a relationship between neuronal firing and saccades (Waitzman et al., 1996; Handel and Glimcher, 1997; Cromer and Waitzman, 2006, 2007). Furthermore, intracellular studies in squirrel monkeys have demonstrated that the activity in a subset of long lead burst neurons in the MRF is tightly coupled to the activity in the intermediate gray layer (SGI) of the superior colliculus (SC; Moschovakis et al., 1988a). There is also evidence that the MRF plays a role in head movements, as reversible lesions of the MRF cause changes in head orientation (Waitzman et al., 2000a). Furthermore, recordings from head-free macaques demonstrate that the discharges of a subset of cMRF neurons are better correlated with the head component of a gaze change than the eye component(Pathmanathan et al., 2006a,b).

Anatomical investigations support a role for the MRF in gaze control. For example, the MRF has been shown to be reciprocally connected with the SC (cat: Edwards and de Olmos, 1976; Graham, 1977; Edwards et al., 1979; May et al., 2002; monkey: Harting, 1977; Cohen and Büttner-Ennever, 1984; Chen and May, 2000; Zhou et al., 2008; Wang et al., 2010). The intra-axonal staining evidence of Moschovakis et al. (1988a, b) is particularly enlightening in this regard. It showed that predorsal bundle (PB) axons projecting from SC provide extensive collaterals within the ipsilateral MRF on their way to their decussation beneath the oculomotor nucleus. They further demonstrated that intracellularly injected reticulotectal neurons within the MRF send axons to terminate either ipsilaterally, or bilaterally, within SGI. We have recently demonstrated that this reticulotectal projection contains both GABAergic and non-GABAergic elements (Wang et al., 2010). There is also evidence that the MRF has downstream projections to the paramedian reticular formation of the pons and medulla (cat: Edwards, 1975; Stanton and Greene, 1981; monkey: Zhou et al., 2008; Perkins et al., 2009). Some of these axons may reach as far as the cervical spinal cord, where head turns are organized (cat: Warren et al., 2008; monkey: Castiglioni et al., 1978; Robinson et al., 1994; Warren et al., 2008; Perkins et al., 2009).

Based on this pattern of connections, and the presence of several populations of neurons that display different patterns of neuronal activity in the cMRF, Waitzman and colleagues have posited the presence of physiologically discrete feedback and feed-forward populations (Cromer and Waitzman, 2006, 2007; Pathmanathan et al., 2006a,b). They suggest the feedback populations may either control the burst activity of saccade-related firing in the SC or provide it with ascending information about gaze-related activity in lower levels of the brainstem. In contrast, the feed-forward pathways may provide an initial stage in the spatio-temporal conversion of the topographically defined gaze vector signal present in the SC into premotor neuron activity suitable to drive motoneurons in the brainstem and spinal cord. However, there is little anatomical evidence to demonstrate whether reticulotectal feedback projections and reticuloreticular feed-forward projections do, in fact, originate from separate MRF populations. Consequently, the focus of the present study was to determine whether the MRF contains neurons whose axons branch and supply both the SC and either the pontine or medullary reticular formation (MdRF). This was accomplished by the use of paired injections of different fluorescent tracers into (1) the SC and (2) either the pontine reticular formation (PRF) or MdRF. Preliminary findings have been reported in abstract form (Perkins et al., 2005).

## MATERIALS AND METHODS

This anatomical study utilized multiple fluorescent neuronal tracer injections in 18 adult cats (*Felix domesticus*) of both sexes. These experiments were conducted in accordance with the NIH policies for humane care and use of laboratory animals under protocols approved by the IACUC at the University of Mississippi Medical Center.

### SURGICAL PROCEDURES

To characterize the pattern and distribution of reticulotectal and reticuloreticular neurons within the MRF of the cat, we made paired injections of different fluorescent tracers: fast blue (FB; Polysciences Inc., Warrington, PA, USA), diamidino yellow (DY; Sigma-Aldrich Corp., St. Louis, MO, USA), and fluorogold (FG; Fluorochrome, LLC., Denver, CO, USA). We initially experimented with different tracer combinations. Four cats had a FB solution deposited in the left SC. In two of these cats, a cocktail of FG and DY was placed into either the PRF or MdRF on the same side. In the other two cats, DY alone was placed into the PRF or MdRF. Reticulotectal neurons labeled robustly with FB were observed. However, the FB signal was so intense, presumably due to the short distance between the MRF and SC, that we worried it might obscure reticuloreticular neurons labeled from the DY injection. We subsequently tested the combination of DY and FG by injecting one of them into left SC and the other in to the rights SC. This combination was found to produce readily visible double labeling.

All fluorescent tracers were dissolved in sterile saline, to give a final concentration of 10% for FG, 4% for FB and 4% for DY. The tracer injection volumes range from 0.1 to 0.5 μl. Cats received unilateral injections of either FG (*n* = 12) or FB (*n* = 4) into the SC. Animals with collicular FG injections received a second injection of DY in the reticular formation, while those with collicular FB injections received either a cocktail of FG and DY (*n* = 2) or DY (*n* = 2). The reticular formation injections were divided between cases with PRF injections (*n* = 6) and MdRF injections (*n* = 6). All these cases received collicular and reticular injections placed on the same side. In four additional animals, FG was placed in the left SC, and DY was placed into the right PRF (*n* = 3) or MdRF (*n* = 1).

Following sedation with isoflurane (1–3%), a catheter was placed in a foreleg vein. Cats were then anesthetized with sodium pentobarbital (up to 35 mg/kg, IV, to effect, supplemented as needed) and placed in a stereotaxic apparatus (Kopf). Atropine sulfate (0.05 mg/kg, IM) helped reduce airway secretions, and dexamethasone (2.5 mg/kg, IV) minimized cerebral edema. Core body temperature was monitored and maintained within normal limits. Aspiration of the medial occipital cortex following a unilateral craniotomy revealed the dorsal surface of the SC. A 1 μl Hamilton syringe attached to a manipulator was visually guided to the SC surface, and then lowered 1.0–1.5 mm into the tissue before making the pressure injection. A second tracer was placed in either the PRF or MdRF based on stereotaxic coordinates (Snider and Niemer, 1961). Specifically, a hole was drilled through the bony tentorium for the PRF injection, or the occipital bone overlying the cerebellum and medulla for the MdRF injection. The syringe needle was angled 26°, tip rostral in the sagittal plane for PRF injections, or 10°, tip medial in the coronal plane for MdRF injections.

We also tested the effectiveness of our techniques on a system known to exhibit branched axons. Moschovakis et al. (1988a) had demonstrated that some cMRF neurons project bilaterally to the SC in the squirrel monkey through the use of intracellular staining. We utilized this reticulotectal model in two additional cats by making a collicular injection of one tracer into the left SC using the methods described above. The coordinate of that injection was then reflected across the midline to direct an injection of the second tracer into the right SC.

Following the last injection, the aspiration defect was filled with hydrated gel foam, and overlying muscle and skin were closed. The wound margins were infused with bupivacaine (0.5–1.0 ml). Each animal received a 60 ml saline, subcutaneous injection for hydration. Animals received an analgesic, Buprenex (0.01 mg/kg), upon recovery from anesthesia. Following a 2 weeks survival period, cats were deeply anesthetized with sodium pentobarbital (50 mg/kg, IP). They were then perfused through the heart with a buffered saline pre-wash, followed by a fixative consisting of 4% paraformaldehyde in 0.1 M, pH 7.2 phosphate buffer (PB).

### TISSUE PREPARATION AND ANALYSIS

The brainstem was blocked in the stereotaxic plane, post fixed in the same fixative over night at 4°C, and then cryoprotected in 30% sucrose PB. It was then frozen and sectioned on a sliding microtome. Sections were cut at 80 μm and retained in serial order. Every third section was mounted on gelatinized slides, air dried, dehydrated, cleared, and coverslipped with non-fluorescing mounting medium. A second one in three series of sections, was mounted and counterstained with cresyl violet to identify brainstem cytoarchitecture.

To facilitate comparison with physiological studies, the cat MRF was divided into a rostral portion located at the level of the InC, termed the piMRF, and a caudal portion, lying behind the piMRF, and extending to the level of the trochlear nucleus, termed the cMRF. The distributions of singly and doubly labeled reticulotectal and reticuloreticular neurons within the piMRF and the cMRF, were plotted at a magnification of 450× using a Leitz Diaplan fluorescence microscope (Leica) equipped with stage optical encoders and MD3 Microscope Digitizer interface controlled by MDPlot software (Accustage, Shoreview, MN, USA) that employed Leica filter cube A, with an excitation range that is wide band UV, and an excitation filter of BP 340–380, and a LP430 barrier filter. With this combination, DY could be seen in the nucleus and FB or FG could be seen in the cytoplasm, simultaneously. The digitized plots were then overlain on drawings obtained from adjacent Nissl-stained sections by use of a BH-2 microscope (Olympus) equipped with a drawing tube. Counts of labeled reticulotectal and reticuloreticular cells were obtained using MDPlot software. Photomicrographs of representative, fluorescently labeled MRF cells were generated with an Eclipse E600 photomicroscope (Nikon) equipped with a Digital DXM1200F color camera (Nikon), by use of MetaMorph analysis software (Molecular Devices, LLC, Sunnyvale, CA, USA). The digitized images were adjusted in Photoshop (Adobe) to appear as close as possible to the visualized image. In this case, a Nikon UV-2E/C filter cube was used, with an excitation wavelength of 340–380 and a barrier filter of 435–485.

## RESULTS

### BILATERAL COLLICULAR INJECTIONS

**FIGURE 1:**
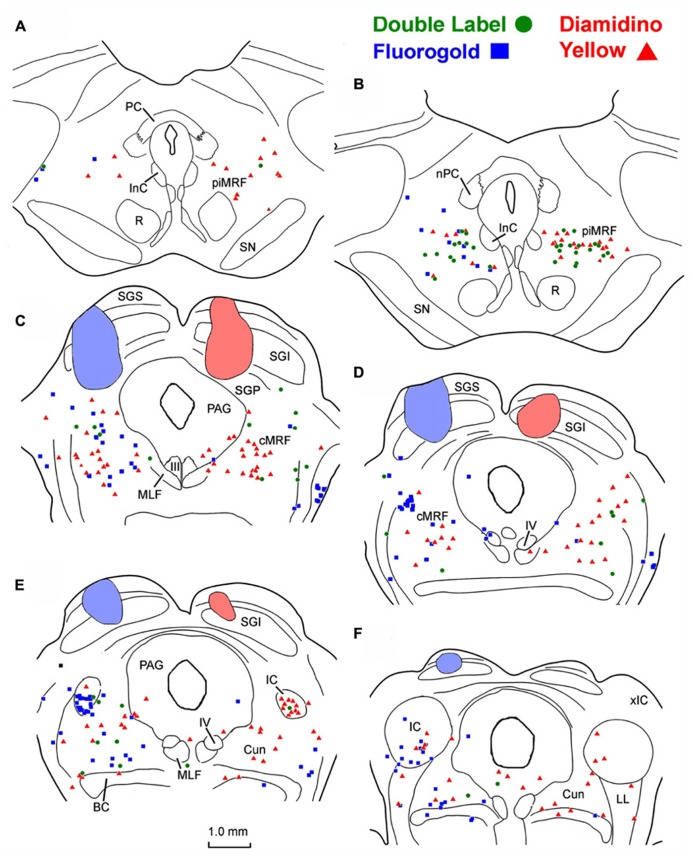
**Injection sites and distribution of labeled ipsilaterally, contralaterally, and bilaterally projecting reticulotectal in MRF.** Cells labeled following a fluorogold injection in the left SC ■ and a diamidino yellow injection into the right SC ▲ are found in both the piMRF **(A,B)** and cMRF **(C,D)**, as well as in the cuneiform nucleus **(E,F)**. Doubly labeled neurons (•) were most common in the piMRF **(A,B)**.

To examine the ability of fluorescent tracers to double label MRF neurons, we utilized the fact this region is known to project bilaterally to the SC. In these cases, a fluorogold (FG) injection was made into the left SC and DY was injected into the right SC. In the example shown (**Figure 1**), the FG injection was located in the lateral SC and heavily involved the intermediate gray layer and those layers superficial to it. The DY injection was located in the medial SC (**Figures 1C–F**). It extended deeper to include the stratum griseum profundum (SGP). Both these injections produced singly labeled cells (FG – blue squares, DY – red triangles here and in subsequent chartings) on both sides of the MRF, in the more caudally located cMRF (**Figures 1C,D**), with a slight ipsilateral predominance. The labeled cell distribution continued further caudally into the cuneiform nucleus (Cun; **Figures 1E,F**). Rostrally, singly labeled cells were observed on both sides in the MRF adjacent to the InC, the piMRF (**Figures 1A,B**), although ipsilaterally projecting reticulotectal cells seemed to predominate. Numerous doubly labeled cells (green circles) were also present in the MRF. These were far more common in the piMRF and in the rostral cMRF.

**FIGURE 2:**
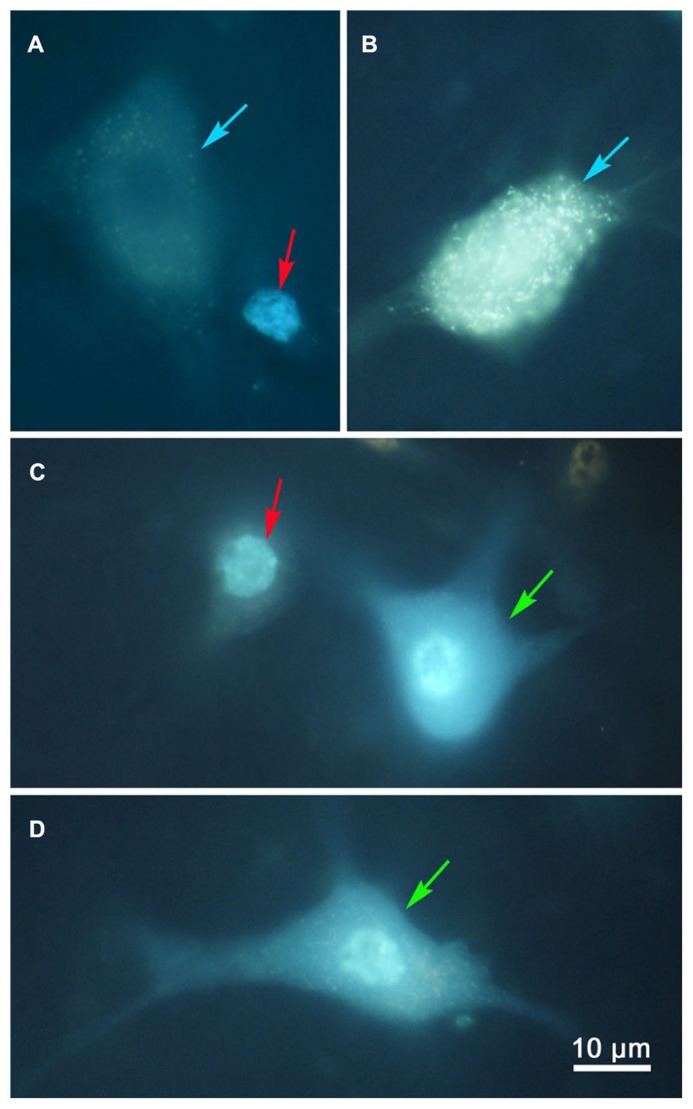
**Photomicrographs of singly (A,B) and doubly **(C,D)** labeled MRF reticulotectal neurons.** Fluorogold produced label in the cytoplasm of the singly labeled cells [**(A,B)**, blue arrows]. Diamidino yellow just labels the nucleus [**(A)**, red arrows]. Doubly labeled neurons show label in both the cytoplasm and nucleus [**(C,D)**, green arrows].

Examples of the labeling produced by these injections are shown in **Figure 2**. Doubly labeled cells (**Figures 2C,D**, green arrows) displayed one tracer in the cytoplasm (FG) and a second tracer in the nucleus (DY). By contrast, the singly labeled cells from the right collicular injection in this case showed only DY labeling of the nucleus (**Figure 2A**, red arrows), and those labeled from the left colliculus showed just FG labeling in the cytoplasm (**Figures 2A,B**, blue arrows). Lipofuscin lightly labeled the cytoplasm of the neuron shown in **Figure 2D**. Based on the results from the dual collicular injection cases, in subsequent combination injections, a 10% FG solution was injected into the SC, and a 4% DY solution was placed into either the PRF or MdRF, in the belief that this pairing of retrograde tracers would facilitate identification and discrimination of any double labeled neurons in the MRF.

### COMBINED COLLICULAR AND PONTINE INJECTIONS

**FIGURE 3:**
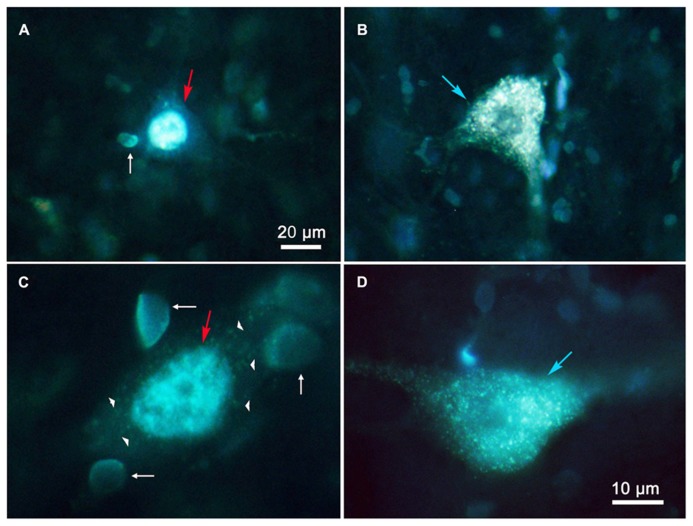
**Photomicrographs of labeled reticulotectal and pontine reticuloreticular neurons in MRF after combined injections of SC and PRF.** **(A,C)** DY labeled reticuloreticular neurons. **(B,D)** FG labeled reticulotectal neurons. The pale fluorescent bodies (white arrows) appear to be glial nuclei. Lipofuscin (arrowheads) is present in the cytoplasm of a DY labeled cell **(C)**. Both **(A,C)** show faint membrane autofluorescence.

**Figure 3** shows photomicrographs of retrogradely labeled MRF neurons following combined injections into the SC and PRF. **Figures 3A,C** show pontine reticuloreticular neurons retrogradely labeled with DY (red arrows) from a PRF injection at low and high magnifications, respectively. The DY labeled neuron displays robust labeling of its nucleus. There is an obvious absence of FG label in the cytoplasm. Apparently the DY can be passed to adjacent glia, resulting in the small, pale fluorescent nuclei noted as satellite labeling in this tissue (**Figures 3A,C** white arrows). **Figures 3B,D** show examples of retrogradely labeled reticulotectal cells (blue arrows) at low and high magnifications, respectively. The cytoplasm of these cells is uniformly filled with FG particles, but the nucleus is devoid of any DY label. Lipofuscin (arrowheads) is also present and fluoresces when exposed to the same wavelengths of illumination. However, it can be distinguished from FG label based on color – it has a yellow or even brown tone, while the fluorogold tends to be silver-white or even silver-blue. In addition, the lipofuscin appears as clumps within the cytoplasm, whereas the FG appears as a fine particulate label uniformly distributed throughout the cytoplasm of the soma and extending into the dendrites.

**FIGURE 4:**
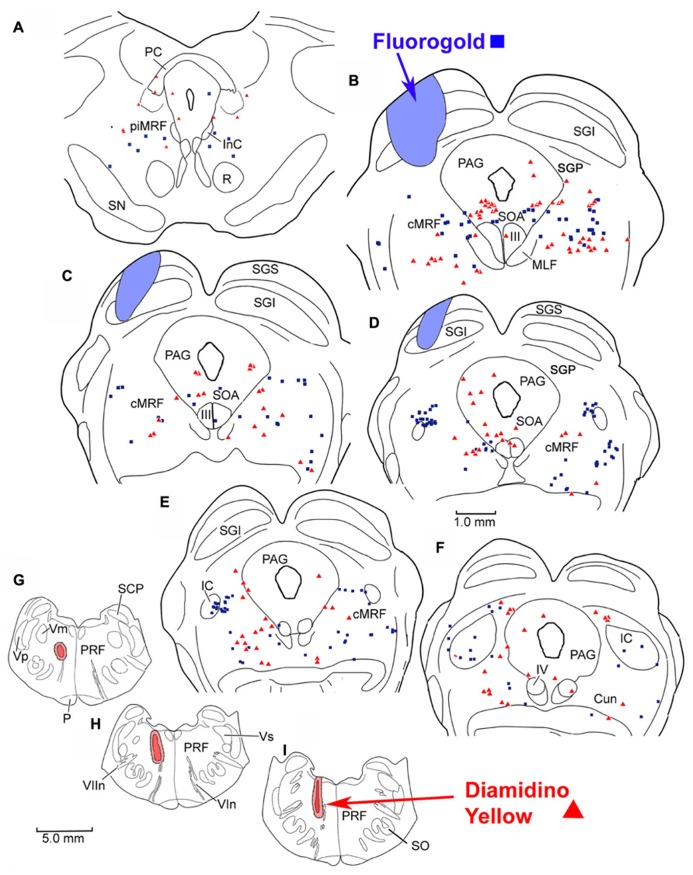
**Injection sites and distribution of labeled reticulotectal neurons ■ and pontine reticuloreticular neurons ▲ in MRF (A–F) following a fluorogold injection in the SC (A–D) and a diamidino yellow injection into the lateral PRF (G–I)**.

**FIGURE 5:**
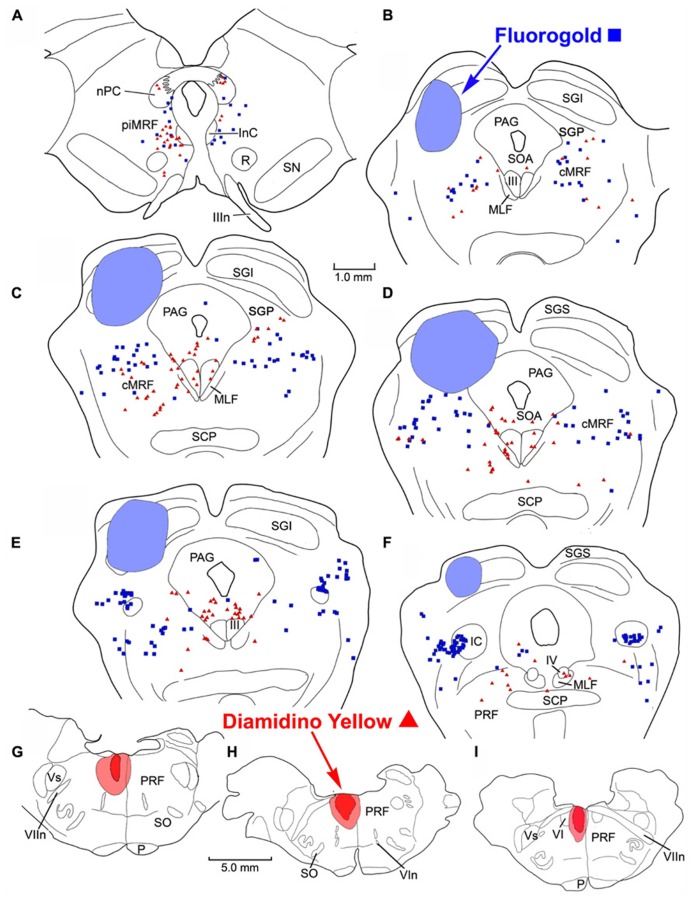
**Distribution of labeled reticulotectal neurons ■ and pontine reticuloreticular neurons ▲ (A–F)** from a fluorogold injection the SC **(A–D)** and a diamidino yellow injection into the medial PRF **(G–I)**.

To investigate the possibility of topography within MRF inputs to distinct regions of the PRF, we made both *lateral* (**Figure 4**) and *medial* (**Figure 5**) injections into the pons. The medial injections included the region termed the paramedian pontine reticular formation (PPRF) which contains premotor saccadic burst neurons. **Figure 4** shows a case where FG was confined to the SC, but involved portions of all its layers (**Figures 4B–D**). The caudal, medial and lateral edges of the SC were spared. A second injection of DY in this same animal was directed laterally into the PRF on the same side (**Figures 4G–I**). The injection site was narrowly focused, and restricted to the area of the PRF rostral to the abducens nucleus, about 1.5 mm lateral to the midline.

The pattern and distribution of labeled MRF neurons from the collicular FG injection and the *lateral* PRF DY injection were charted onto a rostrocaudally ordered series of sections (**Figures 4A–F**). Obvious double labeled neurons were not observed within the MRF following this pair of injections, although cells were occasionally encountered that could not be classified because they exhibited equivocal nuclear labeling. FG labeled reticulotectal cells (blue squares) were observed bilaterally within the cMRF (**Figures 4B–E**). These FG labeled reticulotectal cells were spread throughout mediolateral extent of the cMRF on both sides. At rostral levels of the MRF, within the piMRF (**Figure 4A**), FG cells were also present bilaterally, although those observed ipsilaterally were more densely labeled. A cluster of FG labeled cells was concentrated at the rostral pole of the inferior colliculus (IC; **Figure 4D**). Similar to the labeled reticulotectal neurons, DY labeled pontine reticuloreticular neurons (red triangles) were also distributed bilaterally within the cMRF (**Figures 4B–E**). However, they were mainly found ipsilaterally in piMRF (**Figure 4A**). The DY labeled reticuloreticular cells were also observed in both the medial and lateral portions of the cMRF all along its rostrocaudal axis (**Figures 4B–E**). A few cells labeled with DY or FG extended into the Cun (**Figure 4F**).

The injection sites and the distribution of labeled neurons in MRF following combined injections into the left SC and the *medial* PRF are shown in **Figure 5**. The collicular injection involved portions of all SC layers (**Figures 5B–F**). In this case, the tracer spread extended into the dorsolateral portion of the periaqueductal gray (PAG; **Figure 5D**) and slightly below the ventral boundary of the colliculus (**Figures 5B–E**). The DY injection was directed into the PRF, on the same side. It was focused in the area of the PRF that extends rostrally and medially from the level of the abducens nucleus (**Figures 5G–I**). It lay adjacent to the midline, with a very small portion extending across the midline. Obvious double labeled neurons were not observed in MRF. FG labeled reticulotectal cells were observed bilaterally, throughout the rostrocaudal extent of the cMRF (**Figures 5B–E**). They were spread across the mediolateral extent of the cMRF on both sides. FG labeled cells were also observed bilaterally in the piMRF (**Figure 5A**), and were also found in and ventral to the nuclei of the posterior commissure (nPC). DY labeled pontine reticuloreticular cells showed a pattern similar to that of reticulotectal neurons. They were also observed bilaterally throughout the rostrocaudal and mediolateral extent of the cMRF with an ipsilateral predominance (**Figures 5B–E**). In the piMRF, DY labeled reticuloreticular neurons were found ipsilaterally, and were concentrated medially (**Figure 5A**).

### COMBINED COLLICULAR AND MEDULLARY INJECTIONS

**FIGURE 6:**
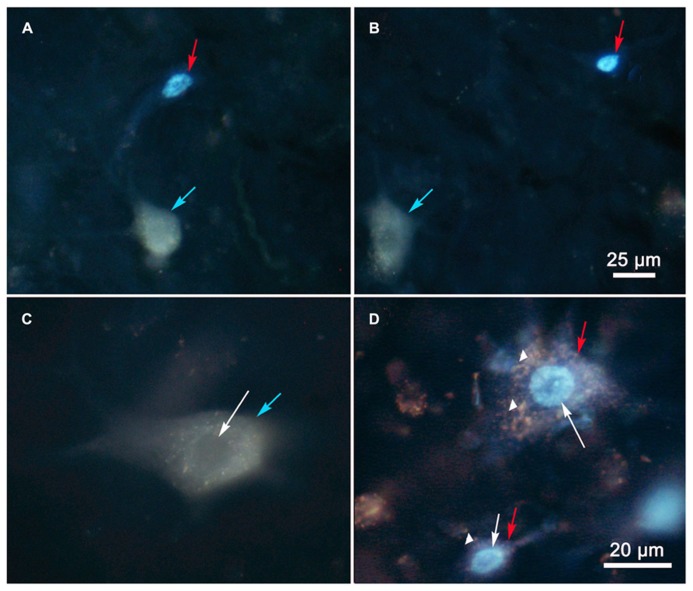
**Photomicrographs of labeled reticulotectal and medullary reticuloreticular neurons in MRF after paired injections into SC and MdRF.** **(A,B)** The close relationship of DY labeled medullary reticuloreticular neurons (red arrows) and FG labeled reticulotectal neurons blue arrows) show spatial overlap between medullary reticuloreticular and reticulotectal neurons in the MRF. Higher magnification **(C)** of a FG labeled reticulotectal neuron (blue arrow) shows the nucleus (white arrow) is unlabeled compared to the nucleus (white arrow) of the DY labeled cell (red arrow) in **(D)**. The cells in **(D)** are DY labeled neurons that contain lipofuscin (arrowhead) in their cytoplasm.

**Figures 6A–C** display photomicrographs of retrogradely labeled MRF neurons that resulted from combined injections into the SC and MdRF. Examples of FG labeled reticulotectal neurons (blue arrows) and DY labeled medullary reticuloreticular neurons (red arrows) were observed in close proximity within the MRF (**Figures 6A,B**). **Figure 6C** shows an example of a FG labeled reticulotectal neuron (blue arrow) at higher magnification. The cytoplasm of the soma and dendrites is filled with fine uniform particles of FG. The cell nucleus (white arrow) is devoid of label, and is distinguishable from the surrounding labeled cytoplasm. In contrast, **Figure 6D** shows two DY labeled cells (red arrows) whose nuclei display intense fluorescence (white arrows). The lipofuscin in these cells (arrowheads) is clearly distinguishable from fluorogold (compare to **Figure 6C**).

**FIGURE 7:**
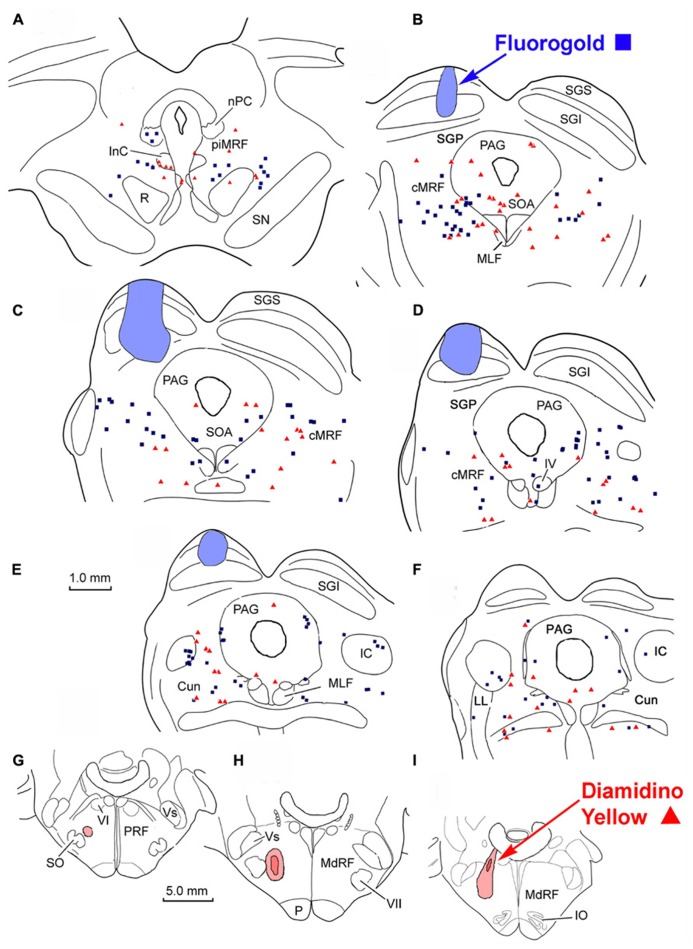
**Distribution of labeled reticulotectal neurons ■ and medullary reticuloreticular neurons ▲ in MRF (A–F)** produced by a FG injection in the SC **(A–E)** and a DY injection into the lateral MdRF **(G–I)**.

Following combined tracer injections into the SC and the *lateral* MdRF (**Figure 7**), retrogradely labeled neurons were again distributed bilaterally within the MRF. In the illustrated example, the FG injection in the left SC included portions of all its layers (**Figures 7B–E**), but did not extend beyond its ventral boundary. The injection spared the more medial and lateral regions of SC, and involved only a small region of SGP. The second tracer, DY, was deposited laterally within the left MdRF of the same animal. The tracer filled the reticular formation immediately medial to the facial nucleus (**Figure 7H**) and was centered about 3 mm lateral to the midline (**Figures 7H,I**). A small amount of tracer extended adjacent to the superior olive, into the PRF. Similar to the pontine injections, there were no obvious double labeled cells noted in the MRF following the medullary injections. Instead, FG labeled reticulotectal cells were found bilaterally in the cMRF as were DY labeled reticuloreticular cells from the MdRF injection (**Figures 7B–D**). The mediolateral distribution of the two populations varied however. Unlike the reticulotectal neurons, which distributed across the mediolateral extent of the cMRF, the majority of the reticuloreticular neurons labeled from the medulla appeared to be restricted to the medial half of the MRF adjacent to the PAG (**Figures 7B–D**). Rostrally, at the level of the piMRF (**Figure 7A**), FG labeled reticulotectal neurons were present bilaterally, with an ipsilateral predominance. DY labeled medullary reticuloreticular neurons were primarily observed ipsilaterally. Additional DY labeled neurons were observed in the PAG (**Figures 7B–F**), the SOA (**Figure 7B**), IC (**Figures 7E,F**), and the Cun (**Figures 7E,F**).

**FIGURE 8:**
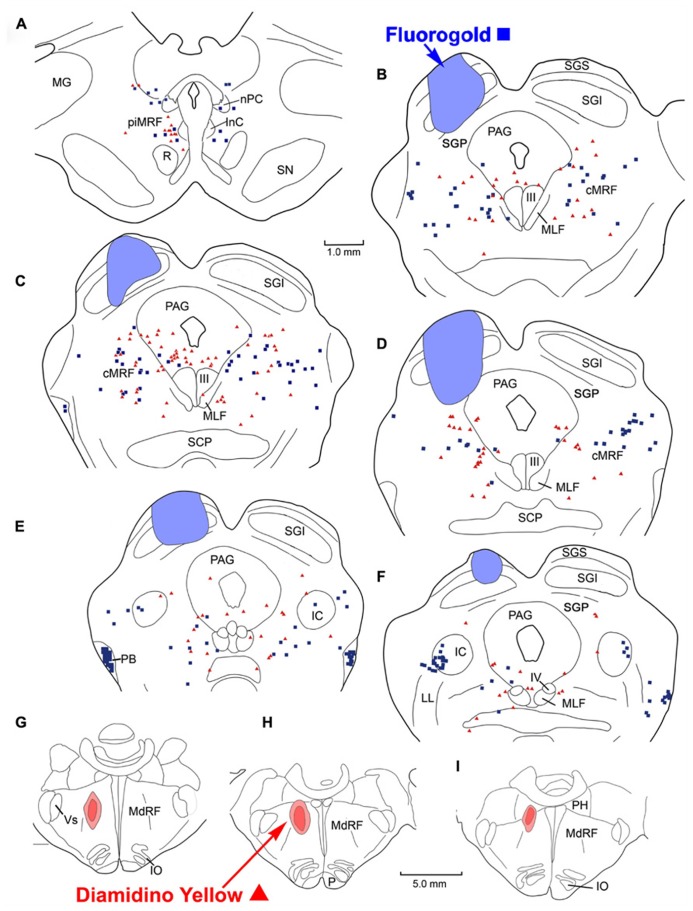
**Distribution of labeled reticulotectal neurons ■ and medullary reticuloreticular neurons ▲ in MRF (A–F)** produced by a FG injection in the SC **(A–E)** and a DY injection into the medial MdRF **(G–I)**.

**Figure 8** illustrates another animal in which the injections were placed into the SC and *medial* MdRF. FG injected into the left SC was largely contained within the boundaries of the colliculus, including all layers (**Figures 8B–F**). However, at the center of the injection site (**Figure 8D**), FG spread beyond SGP to reach the dorsolateral border of the PAG. The injection spared the most medial and lateral aspects of the SC. The second tracer, DY, was directed medially into the left MdRF. DY filled the paramedian portion of the MdRF about 1–2 mm lateral to the midline (**Figures 8G,H**). This included the region of the MdRF, where head movements are evoked by electrical stimulation (Drew and Rossignol, 1990). As with the previous injections, there were no obvious double labeled cells noted in the MRF (**Figures 8A–F**). Caudally, labeled cells from both injections were found bilaterally in the cMRF (**Figures 8B–E**). Rostrally, at the level of the piMRF (**Figure 8A**), FG labeled reticulotectal neurons were present bilaterally, with and ipsilateral preponderance, and DY labeled reticuloreticular neurons were mainly found ipsilaterally. At all levels, the majority of the medullary reticuloreticular neurons appeared to be restricted to the medial half of the cMRF adjacent to the PAG (**Figures 8B–D**).

We examined a limited number of cases in which injections into either the PRF or MdRF were made contralateral to the SC injection instead of ipsilateral. Since there was no obvious difference observed in the distribution of that label compared to the ipsilateral cases, we have not illustrated these results here.

### QUANTIFICATION OF LABELED RETICULOTECTAL AND RETICULORETICULAR NEURONS

The number of retrogradely labeled MRF cells was quantified for three SC-PRF and three SC-MdRF cases. When considering the total number of labeled reticulotectal neurons (*n* = six cases), 55% (mean = 219) were located in the ipsilateral MRF, 45% (mean = 178) were confined to the contralateral MRF. Singly labeled reticuloreticular cells also showed a slight ipsilateral preference with 57% (mean = 107) of labeled reticuloreticular cells in the ipsilateral MRF and 43% (mean = 82) in the contralateral MRF after PRF injections. Similarly, 59% of the MRF reticuloreticular neurons projected to the ipsilateral MdRF, and only 41% projected to the contralateral MdRF. The predominance of ipsilateral labeling was present in all the cases examined. Considerably fewer cells were labeled in the MRF following medullary injections (mean 157) than following collicular (mean = 198) or pontine (mean = 187) injections.

## DISCUSSION

Orchestrating quick head movements to accompany saccadic eye movements requires yoking together a number of brainstem gaze centers. The SC projects by way of the PB to terminate contralaterally in the PPRF, MdRF, and cervical spinal cord (Hashikawa and Kawamura, 1977; Holcombe and Hall, 1981; Huerta and Harting, 1982). This projection represents the main pathway by which the SC effects gaze changes (Harting, 1977; Huerta and Harting, 1982; Hall and May, 1984; Dean et al., 1986; Munoz and Guitton, 1989; May, 2006). In addition, collaterals of the PB axons terminate within the ipsilateral cMRF (Grantyn and Grantyn, 1982; Moschovakis et al., 1988a,b) and help drive a variety of neurons with different gaze signals within this structure (Waitzman et al., 1996; Handel and Glimcher, 1997; Cromer and Waitzman, 2006). The cMRF projects back upon the ipsilateral SC, providing it with an inhibitory feedback signal (Moschovakis et al., 1988b; Appell and Behan, 1990; Chen and May, 2000; Zhou et al., 2008; Wang et al., 2010). The cMRF also projects to the PPRF and MdRF, providing the SC with a trans-cMRF, feed-forward pathway to downstream gaze centers (Edwards, 1975; Stanton and Greene, 1981; Schnyder et al., 1985; Cowie and Holstege, 1992; Cowie et al., 1994; Perkins et al., 2009).

**FIGURE 9:**
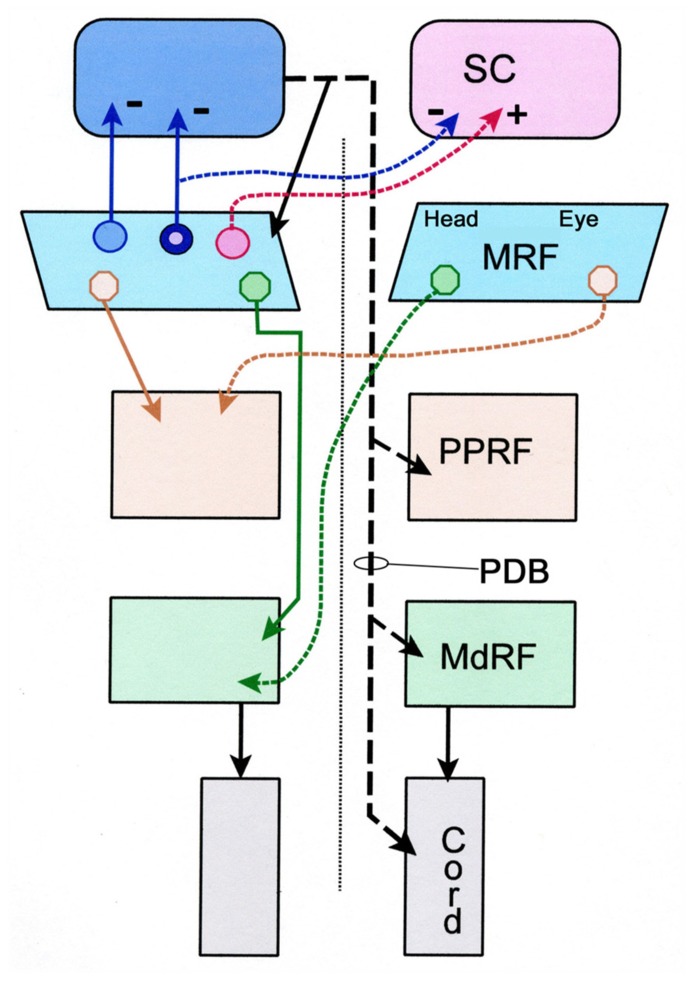
**Schematic diagram showing the pattern of projections of the central portion of the mesencephalic reticular formation (cMRF) to the superior colliculus (SC), and the pontine reticular formation (PRF) and medullary reticular formation (MdRF).** The medial head and the lateral eye regions of the cMRF are indicated for the descending projections. The descending projection from the superior colliculus is the predorsal bundle (PDB). Dashed lines indicate crossed projections. The known excitatory and inhibitory pattern of reticulotectal projections is indicated by + and -, respectively. The reticulotectal neuron projecting to both colliculi has blue cytoplasm and a pink nucleus signifying its appearance when the cytoplasm is labeled with one tracer and the nucleus another.

The main purpose of the present study was to determine whether the projections to the SC, PPRF, MdRF, arise from separate MRF populations (**Figure 9**), which presumably have separate signals, or alternatively originate from a single population, that would ostensibly transmit a unitary signal. The lack of double labeling when paired fluorescent tracers were used to characterize these circuits suggests that the MRF reticulotectal neurons are not the same as the MRF pontine reticuloreticular neurons or the MRF medullary reticuloreticular neurons. Consequently, the feedback pathway to the SC is distinct from either of the feed-forward pathways to the brainstem gaze centers, and these ascending and descending paths likely transmit different signals. This lack of collateralization contrasts with the projections of the SC. One type of tectal neuron projecting in the PB (T-cells) has been shown to project to the contralateral SC via collaterals, as well as to downstream targets (Moschovakis and Karabelas, 1985; Moschovakis et al., 1988a,b).

The present study also provides preliminary data suggesting the piMRF of the cat contains three populations of reticulotectal cells: bilaterally, ipsilaterally, and contralaterally projecting neurons (**Figure 9**). As demonstrated previously (Moschovakis et al., 1988a), a piMRF reticulotectal population projects bilaterally to the SC. The present study suggests that in the cat these cells tend to lie rostrally, mainly in the piMRF. Waitzman et al. (2000b) suggested the piMRF was preferentially involved in the vertical components of gaze change like the adjacent InC. Bilateral projections may help yoke movements of the two eyes in the vertical plane. Moschovakis et al. (1988a) reported two populations of cMRF reticulotectal neurons: those projecting ipsilaterally and those projecting bilaterally. Here we observed a third output that projects just contralaterally. This agrees with evidence from monkeys that the reticulotectal projection of the MRF contains both a bilateral inhibitory projection and a contralateral excitatory one (Wang et al., 2010). It is likely that the number of double labeled neurons would have been even greater if the two injections had been located in similar parts of the SC, instead of one being primarily located in the upper field and the other in the lower field representation.

The results of this study must be weighed in light of technical considerations. With respect to false negative labeling, it is always possible that cells with numerous terminals in one target and few terminals in another target might not display double labeling in these experiments. We occasionally encountered cells heavily labeled with FG, which we suspected might be lightly labeled with DY (**Figure 2B**). In addition, some DY labeled cells displayed diffuse light cytoplasmic label that could have just been non-specific fluorescence, or may have been very light fluorogold labeling (**Figure 3C**). Thus, we can not say there is absolutely no collateral projection. However, the evidence presented here argues that this is not a significant component of the projection. In general, our SC injections for comparing reticulotectal to reticuloreticular labeling covered the majority of the SC, so topography of the reticulotectal projection is unlikely to have been a critical feature in labeling these neurons.

The possibility of false positive labeling due to tracer spread beyond the intended target or labeling of axons of passage must also be considered. The SC has few fibers passing through it and thus, produces minimal fiber-of-passage problems. Furthermore, most collicular injections were done under direct visualization, to improve the accuracy of tracer placement. While, in some cases there was limited tracer spread into the underlying midbrain reticular formation (MRF) or PAG, the pattern of labeling was not noticeably different from cases which spared these structures. MRF projections targeting the cervical cord (Edwards, 1975; Warren et al., 2008) may have been included in the medial medullary injections, but most of these axons lie ventral to our injection sites. Nevertheless, it is likely that they represent collateral targets, since the MdRF and cervical spinal cord both control head movements. Since the descending fibers targeting the MdRF and spinal cord pass through the PRF, injections of this area do pose a potential fiber-of-passage problem. However, since different distributions were observed when the medulla and pons were targeted, this suggests fiber-of-passage labeling is not an overwhelming concern.

### DISTRIBUTION OF RETICULORETICULAR NEURONS

Injections in the PRF of the cat yielded bilateral retrograde labeling in the MRF with an ipsilateral predominance. More medially placed injections, which would presumably involve the PPRF, produced greater numbers of retrogradely labeled cells than did more laterally placed injections. This agrees with the results seen following injections in the peri-abducens reticular formation in the cat (Stanton and Greene, 1981). Furthermore, the chartings from the anterograde study of Edwards (1975) show a bilateral projection from the midbrain reticular formation to the PRF that is denser ipsilaterally, in agreement with our retrograde results. However, similar injections involving the PPRF of the monkey produced labeled cells almost entirely within the ipsilateral cMRF (Perkins et al., 2009), suggesting species differences may be present.

In cats, MdRF injections also produced bilateral retrograde labeling in MRF that exhibited an ipsilateral predominance. In general, labeled medullary reticuloreticular neurons were fewer in number than pontine reticuloreticular neurons. This finding agrees with the caudalward decrease of bilateral anterograde labeling observed by Edwards (1975). However, the retrograde labeling observed following injections of the primate MdRF was entirely ipsilateral (Cowie et al., 1994; Perkins et al., 2009), again suggesting species differences. Physiological data suggests this medial region of the MdRF is closely involved with head movements (cat: Suzuki et al., 1989; Drew and Rossignol, 1990; macaque: Cowie and Robinson, 1994). As the MdRF is believed to organize head movements, it is noteworthy that the MRF reticulospinal projection is entirely ipsilateral in both cat and monkey. Since the MdRF reticulospinal projections are bilateral, it is possible that the bilateral reticuloreticular projections observed here, still end up only influencing activity in the ipsilateral cervical spinal cord due to the pattern of medullospinal projections.

The fact that labeled reticuloreticular neurons are located in the medial MRF following medullary injections suggests that this region of the MRF may be specifically involved in gaze changes that require head movements. This position is supported by previous reports which indicate that MRF cells projecting to the cervical spinal cord also reside in the medial region of the MRF (Holstege and Cowie, 1989; May et al., 2002; Satoda et al., 2002; Warren et al., 2008). However, Cowie and Holstege’s (1992) study of MdRF inputs in the cat does not support this regional specialization. In their study, HRP injections into the midline of the medulla resulted in bilateral retrograde label throughout the MRF. The differences between their results and ours are most likely due to inclusion of the medullary nucleus raphe magnus in the injection site. Indeed, the anterograde tracer experiments (Edwards, 1975) suggest the MRF provides a dense terminal field within this raphe nucleus. It is unlikely that this structure is involved in gaze, so this projection probably represents input from a different set of MRF neurons than those described physiologically in behaving primates by Waitzman et al. (1996).

### FUNCTIONAL IMPLICATIONS OF SEPARATE RETICULOTECTAL AND RETICULORETICULAR POPULATIONS

The apparent absence of double labeled MRF neurons suggests that feedback signals to the SC are distinct from the feed-forward signals sent to either the saccade center in the PRF or the head control center in the MdRF. This finding agrees with that of Moschovakis et al. (1988a). In their limited sample of long lead burst neurons (LLBNs) recovered from the squirrel monkey cMRF, the cells projecting to SC did not display descending collaterals. Scudder et al. (1996) examined the descending axons of piMRF neurons that targeted the MdRF and PRF. Unfortunately, the staining was insufficient to show whether they had collaterals directed to the SC. Our findings suggest they probably do not.

Indeed, this anatomical evidence showing two distinct populations correlates with the physiological evidence. Activity in cMRF reticulotectal neurons is very similar to that of tectal neurons projecting in the PB (Moschovakis et al., 1988a). However, piMRF cells with descending axons characterized by Scudder et al. (1996) show activity that is much less tightly correlated with the saccade. Furthermore, the cMRF and PPRF have cells with similar responses, in that they encode the velocity of the horizontal component of a saccade (Cromer and Waitzman, 2007), suggesting that those cMRF cells which have extracted a velocity signal from the SC input may be the ones that project to the pontine gaze centers. The cMRF also contains neurons whose activity is best correlated with the head component of a gaze shift (Pathmanathan et al., 2006a,b). These would seem to be good candidates for the cells that project to the MdRF. However, no obvious medial distribution was reported for recorded head-related neurons. Future experiments are needed to determine whether the cells projecting to the MdRF and cervical spinal cord are a subset of those projecting to the PRF, as the distribution of the cells labeled from these sites overlaps in the medial MRF. The number of medullary reticuloreticular neurons in the MRF is fewer than the number of pontine reticuloreticular neurons and reticulotectal cells, underlining the idea that the MRF may be more important for eye control, than head control in gaze.

## AUTHOR CONTRIBUTIONS

All three authors contributed to the surgical procedures and analysis of the results for the experiments described in this submission. All three also contributed to the preparation of the figures and the writing of the document.

## Conflict of Interest Statement

The authors declare that the research was conducted in the absence of any commercial or financial relationships that could be construed as a potential conflict of interest.

## Abbreviations

III: oculomotor nucleus;
IIIn: oculomotor nerve;
IV: trochlear nucleus;
VI: abducens nucleus;
VIn: abducens nerve;
VII: facial nucleus;
VIIn: facial nerve;
Vm: trigeminal motor nucleus;
Vp: principal trigeminal nucleus;
Vs: spinal trigeminal nucleus;
BC: brachium conjunctivum;
cMRF: central mesencephalic reticular formation;
Cun: cuneiform nucleus;
DY: diamidino yellow;
FB: fast blue;
FG: fluorogold;
IC: inferior colliculus;
InC: interstitial nucleus of Cajal;
IO: nferior olive;
LL: lateral lemniscus;
MdRF: medullary reticular formation;
MG: medial geniculate nucleus;
MLF: medial longitudinal fasciculus;
MRF: mesencephalic (midbrain) reticular formation;
nPC: nucleus of the posterior commissure;
P: pyramid;
PAG: periaqueductal gray;
PB: parabigeminal nucleus;
PC: posterior commissure;
PH: nucleus prepositus hypoglossi;
piMRF: peri-InC mesencephalic reticular formation;
PPRF: paramedian pontine reticular formation;
PRF: pontine reticular formation;
R: red nucleus;
SC: superior colliculus;
SCP: superior cerebellar peduncle;
SGI: intermediate gray layer of SC;
SGP: deep gray layer of SC;
SGS: superficial gray layer of SC;
SN: substantia nigra;
SO: superior olive;
SOA: supraoculomotor area;
xIC: external nucleus of IC.

## References

[B1] Appell P. P. Behan M. (1990). Sources of subcortical GABAergic projections to the superior colliculus in the cat. *J. Comp. Neurol.* 302 143–158 10.1002/cne.903020111 2086611

[B2] Bender M. B. Shanzer S. (1964). “Oculomotor pathways defined by electrical stimulation and lesion in the brainstem of the monkey,” in *The Oculomotor System* ed. Bender M. B. (New York: Harper and Row) 81–140

[B3] Büttner U. Büttner-Ennever J. A. Henn V. (1977). Vertical eye movement related activity in the rostral mesencephalic reticular formation of the monkey. *Brain Res.* 139 239–252 10.1016/0006-8993(77)90273-6 406969

[B4] Castiglioni A. J. Gallaway M. C. Coulter J. D. (1978). Spinal projections from the midbrain in monkey. *J. Comp. Neurol.* 178 329–346 10.1002/cne.901780208 415074

[B5] Chen B. May P. J. (2000). The feedback circuit connecting the superior colliculus and central mesencephalic reticular formation: a direct morphological demonstration. *Exp. Brain Res.* 31 10–21 10.1007/s002219900280 10759167

[B6] Cohen B Büttner-Ennever J. A. (1984). Projections from the superior colliculus to a region of the central mesencephalic reticular formation (cMRF) associated with horizontal saccadic eye movements. *Exp. Brain Res.* 57 167–176 10.1007/BF00231143 6519224

[B7] Cohen B. Matsuo V. Fradin J. Raphan T. (1985). Horizontal saccades induced by stimulation of the central mesencephalic reticular formation. *Exp. Brain Res.* 57 605–616 10.1007/BF00237847 3979501

[B8] Cowie R. J. Holstege G. (1992). Dorsal mesencephalic projections to pons, medulla, and spinal cord in the cat. Limbic and non-limbic components. *J. Comp. Neurol.* 319 536–559 10.1002/cne.903190406 1619044

[B9] Cowie R. J. Robinson D. L. (1994). Subcortical contributions to head movements in macaques. I. Contrasting effects of electrical stimulation of a medial pontomedullary region and the superior colliculus. *J. Neurophysiol.* 72 2648–2664 7897481 10.1152/jn.1994.72.6.2648

[B10] Cowie R. J. Smith M. K. Robinson D. L. (1994). Subcortical contributions to head movements in macaques. II. Connections of a medial pontomedullary head-movement region. *J. Neurophysiol.* 72 2665–2682 7534824 10.1152/jn.1994.72.6.2665

[B11] Crawford J. D. Cadera W. Vilis T. (1991). Generation of torsional and vertical eye position signals by the interstitital nucleus of Cajal. *Science* 252 1551–1553 10.1126/science.2047862 2047862

[B12] Cromer J. A. Waitzman D. M. (2006). Neurons associated with saccade metrics in the monkey central mesencephalic reticular formation. *J. Physiol.* 570 507–523 10.1113/jphysiol.2005.096834 16308353 PMC1479872

[B13] Cromer J. A. Waitzman D. M. (2007). Comparison of saccade-associated neuronal activity in the primate central mesencephalic and paramedian pontine reticular formation. *J. Neurophysiol.* 98 835–850 10.1152/jn.00308.2007 17537904

[B14] Dean P. Redgrave P. Sahibzada N. Tsuji K. (1986). Head and body movements produced by electrical stimulation of superior colliculus in rats: effects of interruption of crossed tectoreticulospinal pathway. *Neuroscience* 19 367–380 10.1016/0306-4522(86)90267-8 3774146

[B15] Drew T. Rossignol S. (1990). Functional organization within the medullary reticular formation of intact unanesthetized cat. I. Movement evoked by microstimulation. *J. Neurophysiol.* 64 767–781 2230923 10.1152/jn.1990.64.3.767

[B16] Edwards S. B. (1975). Autoradiographic studies of the projections of the midbrain reticular formation: descending projections of nucleus cuneiformis. *J. Comp. Neurol.* 161 341–358 10.1002/cne.901610306 50329 PMC8334145

[B17] Edwards S. B de Olmos J. S. (1976). Autoradiographic studies of projections of the midbrain reticular formation: ascending projection of the nucleus cuneiformis. *J. Comp. Neurol.* 165 417–432 10.1002/cne.901650403 1262539

[B18] Edwards S. B. Ginsburg C. L. Henkel C. K. Stein B. E. (1979). Sources of subcortical projections to the superior colliculus in the cat. *J. Comp. Neurol.* 184 309–330 10.1002/cne.901840207 762286

[B19] Fukushima K. Ohashi O. Fukushima J Kaneko C. R. S. (1995). Discharge characteristics of vestibular and saccade neurons in the rostral midbrain of alert cats. *J. Neurophysiol.* 73 2129–2143 7666128 10.1152/jn.1995.73.6.2129

[B20] Graham J. (1977). An autoradiographic study of the efferent connections of the superior colliculus in the cat. *J. Comp. Neurol.* 173 629–654 10.1002/cne.901730403 864027

[B21] Grantyn A. Grantyn R. (1982). Axonal patterns and sites of termination of cat superior colliculus neurons projecting in the tecto-bulbo-spinal tract. *Exp. Brain Res.* 46 243–256 10.1007/BF00237182 7095033

[B22] Hall W. C. May P. J. (1984). “The anatomical basis for sensorimotor transformations in the superior colliculus,” in *Contributions to Sensory Physiology* Vol. 8 ed. Neff E. E. (New York: Academic Press) 1–34

[B23] Handel A. Glimcher P. W. (1997). Response properties of saccade-related burst neurons in the central mesencephalic reticular formation. *J. Neurophysiol.* 78 2164–2175 9325383 10.1152/jn.1997.78.4.2164

[B24] Harting J. K. (1977). Descending pathways from the superior colliculus: an autoradiographic analysis in the rhesus monkey (*Macaca mulatta*). *J. Comp. Neurol.* 173 583–612 10.1002/cne.901730311 404340

[B25] Hashikawa T. Kawamura K. (1977). Identification of cells of origin of tectopontine fibers in the cat superior colliculus: an experimental study with horseradish peroxidase method. *Brain Res.* 130 65–79 10.1016/0006-8993(77)90842-3 69477

[B26] Holcombe V. Hall W. C. (1981). Laminar origin of ipsilateral tectopontine pathways. *J. Neurosci.* 6 255–260 10.1016/0306-4522(81)90061-0 6164009

[B27] Holstege G. Cowie R. J. (1989). Projections from the rostral mesencephalic reticular formation to the spinal cord. *Exp. Brain Res.* 75 265–279 10.1007/BF00247933 2721608

[B28] Huerta M. F. Harting J. K. (1982). Tectal control of spinal cord activity: neuroanatomical demonstration of pathways connecting the superior colliculus with the cervical spinal cord grey. *Prog. Brain Res.* 57 293–328 6296921 10.1016/s0079-6123(08)64135-7

[B29] King W. M. Fuchs A. F. (1979). Reticular control of vertical saccadic eye movements by mesencephalic burst neurons. *J. Neurophysiol.* 42 861–876 107287 10.1152/jn.1979.42.3.861

[B30] May P. J. (2006). The mammalian superior colliculus: laminar structure and connections. *Prog. Brain Res.* 151 321–378 10.1016/S0079-6123(05)51011-2 16221594

[B31] May P. J Warren S. Chen B. Richmond F. J. R. Olivier E. (2002). Midbrain reticular formation circuitry subserving gaze in the cat. *Ann. N. Y. Acad. Sci.* 956 405–408 10.1111/j.1749-6632.2002.tb02841.x 11960826

[B32] Moschovakis A. K. Karabelas A. B. (1985). Observations of the somatodendritic morphology and axon trajectory of intracellularly HRP-labeled efferent neuron located in the deeper layers of the superior colliculus of the cat. *J. Comp. Neurol.* 239 276–308 10.1002/cne.902390304 4044941

[B33] Moschovakis A. K. Karabelas A. B. Highstein S. M. (1988a). Structure-function relationships in the primate superior colliculus. I. Morphological classification of efferent neurons. *J. Neurophysiol.* 60 232-262 3404219 10.1152/jn.1988.60.1.232

[B34] Moschovakis A. K. Karabelas A. B. Highstein S. M. (1988b). Structure-function relationships in the primate superior colliculus. II. Morphological identity of presaccadic neurons. *J. Neurophysiol.* 60 263–302 3404220 10.1152/jn.1988.60.1.263

[B35] Munoz D. P. Guitton D. (1989). Fixation and orientation control by the tecto-reticulo-spinal system in the cat whose head is unrestrained. *Rev. Neurol.* 145 567–579 2554460

[B36] Pathmanathan J. S. Presnell R. Cromer J. A. Cullen K. E. Waitzman D. M. (2006a). Spatial characteristics of neurons in the central mesencephalic reticular formation (cMRF) of head-unrestrained monkeys. *Exp. Brain Res.* 168 455–470 10.1007/s00221-005-0104-0 16292575

[B37] Pathmanathan J. S. Cromer J. A. Cullen K. E. Waitzman D. M. (2006b). Temporal characteristics of neurons in the central mesencephalic reticular formation of head unrestrained monkeys. *Exp. Brain Res.* 168 471–492 10.1007/s00221-005-0105-z 16292574

[B38] Perkins E. May P. J. Warren S. (2005). Distribution of central mesencephalic reticular formation output neurons projecting to brainstem gaze centers. *Soc. Neurosci. Abstr.* 3 858

[B39] Perkins E. Warren S. May P. J. (2009). The mesencephalic reticular formation as a conduit for primate collicular gaze control: tectal inputs to neurons targeting the spinal cord and medulla. *Anat. Rec.* 288 1310–1329 10.1002/ar.20935 PMC2773470 19645020

[B40] Robinson F. R. Phillips J. O. Fuchs A. F. (1994). Coordination of gaze shifts in primates: brainstem inputs to neck and extraocular motoneurons pools. *J. Comp. Neurol.* 346 43–62 10.1002/cne.903460104 7962711

[B41] Satoda T. Matsumoto H. Zhou L. Rose P. K Richmond F. J. R. (2002). Mesencephalic projections to the first cervical segment in the cat. *Exp. Brain Res.* 144 397–413 10.1007/s00221-002-1047-3 12021821

[B42] Schnyder H. Reisine H. Hepp K. Henn V. (1985). Frontal eye field projections to the paramedian pontine reticular formation traced with wheat germ agglutinin in the monkey. *Brain Res.* 329 151–160 10.1016/0006-8993(85)90520-7 3978438

[B43] Scudder C. A. Moschovakis A. K. Karabelas A. B. Highstein S. M. (1996). Anatomy and physiology of saccadic long-lead burst neurons recorded in the alert squirrel monkey. I. Descending projections from the mesencephalon. *J. Neurophysiol.* 76 332–352 10.1152/jn.1996.76.1.332 8836229

[B44] Snider R. S, Niemer W. T. (1961). *A Stereotaxic Atlas of the Cat Brain*. Chicago: University of Chicago Press

[B45] Stanton G. B. Greene R. W. (1981). Brain stem afferents to the peri-abducens reticular formation (PARF) in the cat. *Exp. Brain Res.* 44 419–426 10.1007/BF00238834 7308356

[B46] Suzuki S. S. Siegel J. M. Wu M. F. (1989). Role of pontomedullary reticular neurons in horizontal head movements: an ibotenic acid lesion study in the cat. *Brain Res.* 484 78–93 10.1016/0006-8993(89)90350-8 2713704 PMC9150860

[B47] Waitzman D. M. Silakov V. L. Cohen B. (1996). Central mesencephalic reticular formation (cMRF) neurons discharging before and during eye movements. *J. Neurophysiol.* 75 1546–1572 8727396 10.1152/jn.1996.75.4.1546

[B48] Waitzman D. M. Silakov V. L. DePalma-Bowles S. Ayers A. S. (2000a). Effects of reversible inactivation of the primate mesencephalic reticular formation. I. Hypermetric goal-directed saccades. *J. Neurophysiol.* 83 2260–2284 10758133 10.1152/jn.2000.83.4.2260

[B49] Waitzman D. M. Silakov V. L. DePalma-Bowles S. Ayers A. S. (2000b). Effects of reversible inactivation of the primate mesencephalic reticular formation. II. Hypometric vertical saccades. *J. Neurophysiol.* 83 2285–2299 10758134 10.1152/jn.2000.83.4.2285

[B50] Wang N. Warren S. May P. J. (2010). The macaque midbrain reticular formation sends side-specific feedback to the superior colliculus. *Exp. Brain Res.* 201 701–717 10.1007/s00221-009-2090-0 19940983 PMC2840059

[B51] Warren S. Waitzman D. M. May P. J. (2008). Anatomical evidence for interconnections between the central mesencephalic reticular formation and cervical spinal cord in the cat and macaque. *Anat. Rec.* 291 141–160 10.1002/ar.20644 PMC2859179 18213702

[B52] Zhou L. Warren S. May P. J. (2008). The feedback circuit connecting the central mesencephalic reticular formation and the superior colliculus in the macaque monkey: tectal connections. *Exp. Brain Res*. 189 485–496 10.1007/s00221-008-1444-3 18553075 PMC2859182

